# Blockchain-Assisted Reputation Management Scheme for Internet of Vehicles

**DOI:** 10.3390/s23104624

**Published:** 2023-05-10

**Authors:** Qian Liu, Junquan Gong, Qilie Liu

**Affiliations:** 1 School of Communication and Information Engineering, Chongqing University of Posts and Telecommunications, Chongqing 400065, China; qianliu@cqupt.edu.cn (Q.L.); s200131302@stu.cqupt.edu.cn (J.G.); 2 Key Laboratory of the Ministry of Education on Mobile Communications Technology, Chongqing 400065, China; 3 Chongqing Key Laboratory of Mobile Communications Technology, Chongqing 400065, China; 4 Post Doctoral Research Workstation of Engineering Research Center of Mobile Communications, Ministry of Education, Chongqing 400065, China

**Keywords:** IoV, MEC, data sharing, reputation management, subjective logic trust model, blockchain

## Abstract

With the rapid development of Internet of Vehicles (IoV), particularly the introduction of Mobile Edge Computing (MEC), vehicles can efficiently share data with one another. However, edge computing nodes are vulnerable to various network attacks, posing security risks to data storage and sharing. Moreover, the presence of abnormal vehicles during the sharing process poses significant security threats to the entire network. To address these issues, this paper proposes a novel reputation management scheme, which proposes an improved multi-source multi-weight subjective logic algorithm. This algorithm fuses the direct and indirect opinion feedback of nodes through the subjective logic trust model while considering factors such as event validity, familiarity, timeliness, and trajectory similarity. Vehicle reputation values are periodically updated, and abnormal vehicles are identified through reputation thresholds. Finally, blockchain technology is employed to ensure the security of data storage and sharing. By analyzing real vehicle trajectory datasets, the algorithm is proven to effectively improve the differentiation and detection rate of abnormal vehicles.

## 1. Introduction

In the era of intelligence, Internet of Vehicles (IoV) technology has gained significant attention as it seamlessly integrates with various industries such as automotive, electronics, information and communication, and road transportation [1]. A vast number of internet-connected vehicles can unlock numerous new application values; however, they may also contribute to increased traffic congestion and accidents simultaneously. To address these challenges, experts have proposed Intelligent Transportation Systems (ITS) [2]. As a vital branch of IoT technology applied in the transportation sector, IoV serves as a critical component of future ITS [3]. The IoV can unify intelligent vehicles with public infrastructure, sensors, computing nodes, pedestrians, and other system elements in the surrounding environment. This enhances the safety of all road users through a comprehensive information-exchange platform between vehicles and heterogeneous networks, ultimately fostering a higher quality public environment and space [4].

Numerous applications in the IoV, such as image-assisted navigation, intelligent driving, and gaming entertainment, require real-time computation and the storage of large amounts of data [5]. However, owing to the limited computing resources of vehicles and the long latency of cloud computing, the IoV’s application and development face significant challenges [6]. To address these issues and challenges, researchers have introduced Mobile Edge Computing (MEC) technology into IoV, forming a new network paradigm called Vehicular Edge Computing (VEC) [7]. MEC is a novel computing technology that shifts data processing and analysis tasks from central servers to edge devices on a network, thereby reducing the overall communication and computing latency [8]. By deploying servers near users, MEC provides abundant network resources for resource-constrained vehicles, minimizing the computational latency of vehicle applications and enhancing the computing and storage capabilities of the IoV [9,10]. However, vehicles must offload data and computing tasks to edge nodes before receiving processing results. This data often carries sensitive information, such as the vehicular driving status and location, which may be leaked or tampered with during the process, posing severe threats to vehicle safety [11]. Additionally, some abnormal vehicles in the VEC network may provide irrelevant or erroneous information to other vehicles due to defective components or malicious intent during information sharing [12], thereby posing significant security risks to the network. Therefore, the efficient identification of unreliable vehicles in a network has become an essential research topic in VEC [13].

To address these challenges, the IoV requires a technical solution that ensures the authenticity and integrity of data storage while providing secure and trustworthy data sharing and transaction platforms. Additionally, an accurate calculation of vehicle reputation values is urgently required to differentiate between normal and unreliable vehicles during the sharing process.

## 2. Related Work

Numerous studies have focused on trust issues in data sharing and transactions in the IoV and have analyzed and modeled them. To detect abnormal vehicle nodes, various management schemes and trust models can be employed to assess the reputation of vehicle nodes in the network [14], determining the authenticity of the message content based on the message’s reliability.

References [15,16] proposed to update and manage vehicle reputation levels through trusted authoritative nodes, which then determined whether a vehicle node could access the network. Vehicles must query central nodes for the reputation of other vehicles to evaluate the credibility of the messages. Reference [17] designed a reliable cooperative download reputation system to promote cooperation and penalize malicious vehicles. However, these centralized reputation management schemes face challenges such as single-point failures, high latency, and data leakage, making them unsuitable for the distributed network architecture of VEC.

Blockchain technology with its decentralized, tamper-proof, secure, and traceable features has been utilized for VEC scenarios [18]. With the immutability of distributed ledgers, blockchain allows for the establishment of trust relationships in a decentralized manner among untrusted entities. Therefore, using blockchain to assist VEC networks promotes information transparency among vehicles and ensures the secure storage and sharing of data [19,20]. Additionally, smart contract technology in the blockchain provides decentralized and reliable automated transactions among vehicles. Reference [21] exploited edge computing, blockchain, and a Bayesian network to quickly identify fraudulent messages while considering parameters such as pre-traffic probability, traffic event period, vehicle honesty, and the number of events provided by vehicles. Reference [22] proposed a decentralized and trustworthy data sharing management system for the VEC network using consortium blockchain and smart contracts to enable vehicles to validate message credibility from their neighbors and generate reputation ratings. References [23,24] proposed blockchain-based vehicle-reputation computation methods. Reference [23] used the consortium blockchain and considered direct and indirect opinion sources to calculate vehicle reputation values based on beta and exponential distributions. Reference [24] also employed a Bayesian inference model based on a blockchain vehicle network to validate messages received from neighboring vehicles, generating binary positive and negative ratings for message sources, and calculating vehicle reputation based on weighted distances and events.

However, the aforementioned studies only considered simple binary logic and weighting to obtain the final reputation value, which cannot cope with the complexity of real-world events. The Subjective Logic (SL) trust model [25,26] is a widely applied mathematical tool for modeling vehicle reliability because it can quantify trust, doubt, and uncertainty [27] and consider the trustworthiness of opinion sources. By combining the probability theory and logical reasoning, SL models represent the objective truth of events more accurately than simple binary logic.

Blockchain-assisted reputation-management methods for VEC networks based on the SL trust model and their evolving models have garnered attention from researchers. Reference [28] introduced the SL trust model into mobile ad hoc networks and calculated fused direct and indirect reputation values by considering weights and weight-transfer formulas. Reference [29] introduced the SL trust model into fog computing for node-to-node trust computation and proposed a peer-to-peer bidirectional SL trust management method that allows service requesters to verify the trustworthiness of service providers and vice versa. Reference [30] utilized blockchain and smart contracts to ensure fair task and mitigate security attacks, and further created a trustfulness assessment mechanism using a subjective logic-based metric that calculates the likelihood of task success. References [31,32] both proposed a Three-weight Subjective Logic (TWSL) algorithm based on VEC, considering factors such as vehicle familiarity, timeliness, and trajectory similarity for a more accurate vehicle reputation value computation. Reference [33] further presented a credit scheme based on a four-weight SL trust model that considered factors such as node resource availability, event validity, familiarity, and timeliness. Reference [34] proposed a Multi-Weight Local Opinions for Subjective Logic algorithm for IoV, which considers factors such as the rate of experiences, recent experiences, experience effects, and other variables to calculate and update reputation. Reference [35] proposed an algorithm based on a three-value SL trust model to evaluate vehicle reputation values by considering factors such as vehicle historical cycle reputation values, feedback party reputation values, and familiarity with updating vehicle reputation values.

To summarize, the majority of existing studies on reputation management methods based on blockchain and the SL trust model do not address the simplicity and singularity of opinion sources, and do not consider the fusion of multiple opinion sources. While some schemes incorporate multi-hop indirect opinions, their validation is based on simulation software rather than real datasets to verify the algorithm’s time complexity and scheme effectiveness. Additionally, the majority of the studies only apply various weights to final opinions, without considering the feedback of the shared events themselves as dynamic weights.

This paper focuses on the background of reputation calculation in IoV with the assistance of blockchain technology, specifically exploring the issue of reputation value calculation in the process of vehicle information sharing. The main research objectives are as follows:

1. We propose a reputation management scheme for data sharing in the IoV networks using a consortium blockchain and MEC technology.
2. We present a vehicle information credibility assessment scenario that maximizes the relevance and reliability of feedback while preventing misbehaviors during the data sharing process.
3. We formulate a data sharing algorithm for IoV networks that incorporates familiarity, timeliness, trajectory similarity, and event validity weights to derive direct and indirect combined opinions, followed by performance evaluation through simulations.

The rest of the research article is organized as follows. Section 2 presents the literature review of the state-of-the-art works and the main contributions of this paper. Section 3 describes the system model and the specific process involved. Section 4 elaborates on the computational process of the proposed scheme. Simulation results and discussion are provided in Section 5. Lastly, Section 6 provides the conclusions.

## 3. The Framework of Blockchain-Assisted Reputation Management Scheme for IoV

### 3.1. System Model

**Figure 1:**
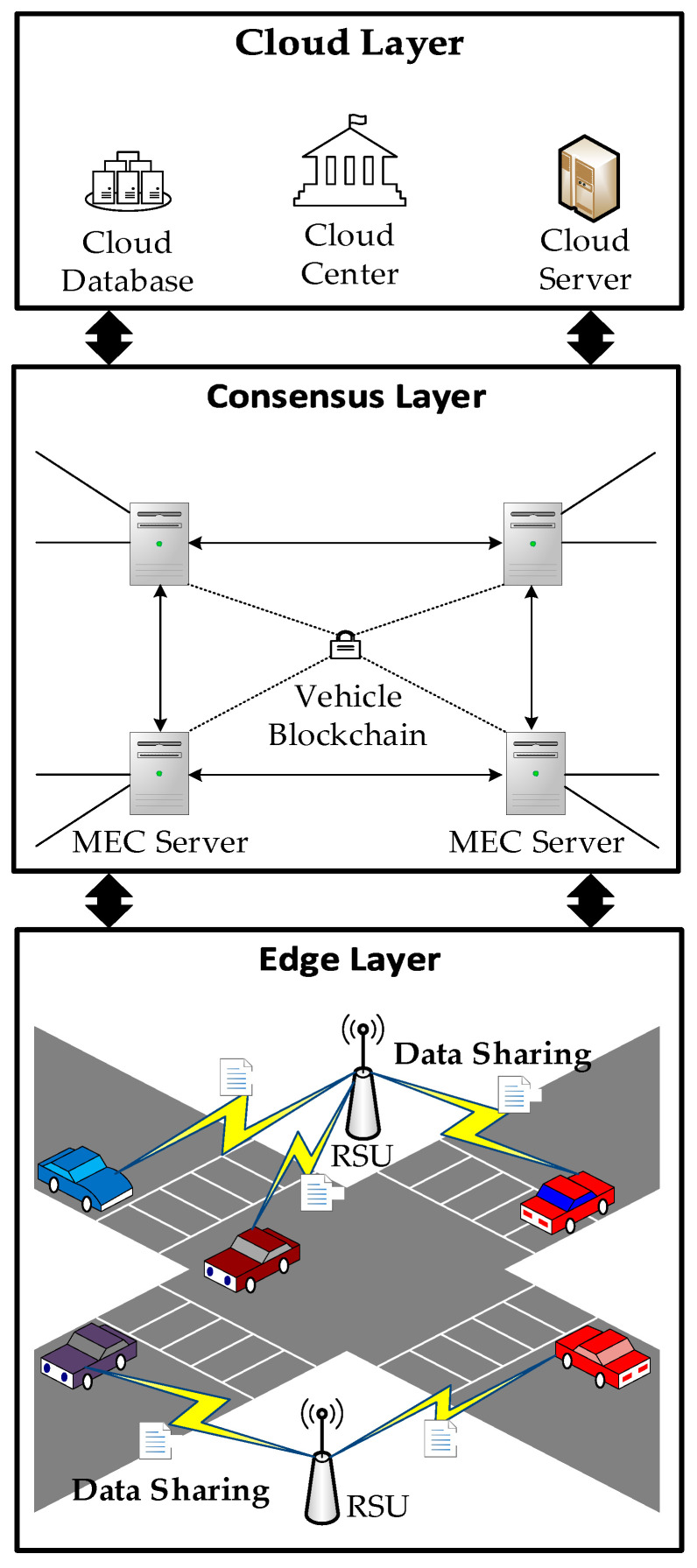
System model.

In a consortium blockchain, a distributed ledger is created, audited, and shared by multiple authorized nodes, offering cost effectiveness and better suitability for VEC network scenarios [36]. Therefore, the system model in this study is based on a consortium blockchain and VEC consisting of the edge, consensus, and cloud layers. The system model is shown in Figure 1:

The cloud layer centrally manages cloud servers, data centers, etc., and is primarily responsible for complex data processing, analysis, optimization, storage, and other functions. It is also responsible for identity verification and the authorization of the consortium blockchain. A central cloud can permanently store large amounts of data and perform complex delay-tolerant computing tasks for vehicles. Frequently used data and time-sensitive tasks in vehicles can be completed using a consensus layer.

In the consensus layer, a vehicle edge cluster is formed by connecting Roadside Units (RSUs) deployed along the road to the MEC servers and interconnecting them. This formation enables real-time data synchronization among the MEC servers within the cluster, thus addressing synchronization issues and vehicle concurrency problems in the network. Each vehicle communicates with the nearest RSU to access the local vehicle-edge cluster. The layer’s primary focus is on aggregating and managing data and reputation values, selecting and allocating resources, and transmitting data to the central cloud via wired connections when necessary.

In the edge layer, vehicles equipped with onboard units can access services by communicating with the RSUs. Onboard units perform simple computations, collect local traffic condition data from sensor devices, and upload the data to the consensus layer through the RSUs. This layer is primarily responsible for data generation, transmission, and sharing.

### 3.2. Sharing Process

**Figure 2:**
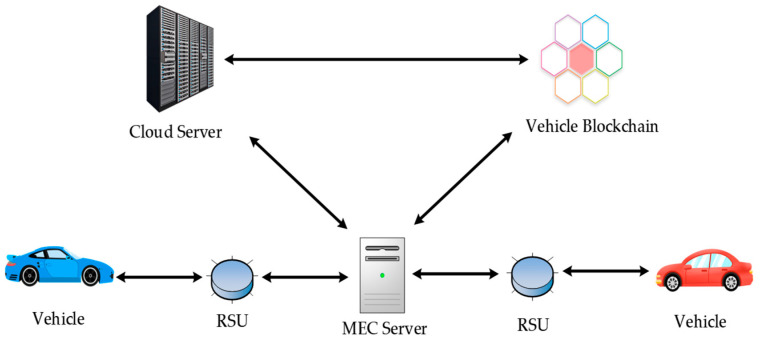
Flow diagram.

To reduce the costs associated with establishing and operating a blockchain, this study employs consortium blockchain technology to construct a vehicle blockchain and utilizes smart contract technology to facilitate distributed data storage and secure data sharing. The process schematic of this approach is illustrated in Figure 2.

#### 3.2.1. Network Initialization

Initially, the RSUs at the edge layer accept the vehicle registration information and send the data to the MEC servers, which collect and organize the data before forwarding them to the central cloud for review.

Subsequently, the central cloud obtains the vehicle’s electronic identity and legal documents from the vehicle management department, based on which it conducts audits and registrations.

Finally, the approved vehicle information is added to the certificate list. The central cloud issues identity certificates and public–private key pairs for the blockchain to the corresponding vehicles and distributes the latest permit list to all MEC servers and RSUs.

#### 3.2.2. Information Upload

Mobile vehicles are equipped with computing and storage resources, as well as various sensors, including LiDAR, millimeter-wave radar, GPS terminals, and high-definition cameras. These vehicles continuously transmit trajectory data and environmental information, such as latitude and longitude information, road conditions, weather conditions, and parking space utilization rates, to RSUs, which package this data into “blocks.” These “blocks” are eventually sorted and stored in the consortium blockchain by MEC servers, and can be provided to other users in the system for browsing and downloading. To protect user security and privacy, data should be anonymized, encrypted, and accompanied by the digital signature of the data provider.

#### 3.2.3. Information Sharing

Initially, data requesters download shared data blocks within a specific range from the vehicle blockchain via the RSUs and search for the data they are interested in using an information index. Based on their actual needs and the data provider’s reputation, data requesters select the best provider and generate a smart contract that reflects their needs and the data provider’s reputation.

Upon receiving the request, the RSUs first verify the identity of the data requester and send information such as the requester’s public key to a nearby MEC server. The MEC server then verifies the data requester’s certificate and locates the requested shared information within the vehicle blockchain. The information is encrypted using the data requester’s public key and then sent directly to the requester.

After obtaining the shared data, the data requester automatically pays the provider according to the contract and generates a record of the data-sharing event. This record, in the form of a data block, is added to the blockchain.

Finally, at the end of the transaction, the data requester provides feedback to the system based on the actual data situation, indicating whether the event was positive or negative. Based on this feedback, the system periodically updates the reputation values. The MEC server packages all transactions and updates reputation values into a block, which is then broadcast to the network to reach a consensus. After the PBFT consensus process, the block is recorded in the blockchain.

Based on the PBFT consensus mechanism, all participating nodes have equal status, with one node selected as the primary node and the remaining nodes serving as backup nodes. The PBFT algorithm comprises three stages: pre-preparation, preparation, and commit. In each stage, when a node receives replies from more than two/three of the other nodes, it proceeds to the next stage.

## 4. The MSMWSL Algorithm for Reputation Management

### 4.1. Direct Opinion Combination

Based on the SL model, by considering the direct opinion weights between shared vehicle nodes such as familiarity, timeliness, and trajectory similarity, a more accurate quantification of the differences in subjective opinions between vehicle nodes can be achieved, resulting in a direct opinion combination.

#### 4.1.1. Direct Opinion Three Weights

1. Familiarity

Familiarity measures the degree of familiarity between vehicles; a higher familiarity implies a higher interaction frequency and more prior information, resulting in more reliable feedback. In this study, the familiarity between vehicle nodes is the ratio of the total interaction count between vehicle nodes within a certain period to the average interaction count of other vehicle nodes, denoted as $F_{i\to j}$:

(1) $$\left\{ \begin{array}{l} F_{i\to j}=N_{i\to j}/\bar{N_{j}}\\ \bar{N_{j}}=\frac{1}{N}\sum_{m\in M}N_{m\to j} \end{array},\right.$$

where i and j represent data users and data providers, $N_{i\to j}$ denotes the total number of information interactions between vehicle i and j within a specific time window, $\bar{N_{j}}$ represents the average interaction count of vehicle j with other vehicle nodes, N denotes the total number of vehicles j is interacting with, and M represents the set of vehicles that have interacted with the vehicle node.

2. Timeliness

Newly uploaded data are emphasized for information sharing. This study defines the timeliness of direct opinions from vehicle nodes to vehicle nodes denoted by $TIM_{i\to j}$:

(2) $$TIM_{i\to j}=\eta {\left(t_{i}-t_{j}\right)}^{-\varepsilon },$$

where η and ε are predefined parameters for adjusting the timeliness, $t_{i}^{}$ represents the time point when the data user i completes the transaction, and $t_{j}^{}$ represents the time point when the data provider j uploads shared information.

3. Trajectory Similarity

Trajectory similarity measures the similarity between the driving states of the data user and the data provider. In this study, similarity is calculated based on the velocity and direction of the trajectory data in the continuous trajectory segments covered by the onboard sensors of both vehicles during sharing, denoted as $SIM_{i\to j}$:

(3) $$SIM_{i\to j}=\tau_{1}S_{spd}+\tau_{2}S_{dirc},$$

where $S_{spd}$ represents the velocity similarity of the trajectory; $S_{dirc}$ represents the direction similarity of the trajectory; and their weights in the trajectory similarity are $\tau_{1}$ and $\tau_{2}$, respectively, with $\tau_{1}+\tau_{2}=1$.

The similarities of velocity and direction are calculated using the cosine similarity formula:

(4) $$S_{j}^{i}=\sum_{k=1}^{n}c_{k}^{i}c_{k}^{j}/(\sqrt{\sum_{k=1}^{n}{\left(c_{k}^{i}\right)}^{2}}\sqrt{\sum_{k=1}^{n}{\left(c_{k}^{j}\right)}^{2}})$$

where $c_{k}^{i}$ represents the velocity or direction in the trajectory of vehicle node i; $c_{k}^{j}$ represents the velocity or direction in the trajectory of vehicle node j; and k represents the number of trajectory points in a similar trajectory segment during the information sharing process between the two vehicles.

4. Direct Opinion Weights

The final direct weight is obtained by taking the weighted average of the three factors in from Equation (1) to Equation (3):

(5) $$\delta_{i\to j}=\rho_{1}F_{i\to j}+\rho_{2}TIM_{i\to j}+\rho_{3}SIM_{i\to j}$$

where the direct opinion weight parameters are $\rho_{1}$, $\rho_{2}$, $\rho_{3}$, with $\rho_{1}+\rho_{2}+\rho_{3}=1$.

#### 4.1.2. Event Validity Based on Growth Curve Function

1. Growth Curve Function

The growth curve function is a class of mathematical functions used to describe the developmental process of things. Generally, the development process is similar to the biological development process, which includes three stages: initiation, development, and maturity, with different development speeds at each stage. In the initial stage, the change is slow; in the development stage, the change accelerates; and in the maturity stage, the change slows again. The curve of the development process drawn according to the development rules of these three stages is usually called a growth curve or logistic growth curve. This curve has been widely used to describe and predict various fields of technical and economic development. There are many mathematical growth curve models, among which the Gompertz and Pearl models are the most widely used. In reference [37], the Gompertz model was employed to calculate and update the reputation values. In contrast, reference [38] utilized the Pearl model, incorporating the difference between positive and negative events for reputation value calculation. Consequently, this study refers to these publications and adopts a weighted average of negative event counts in conjunction with the Pearl model for reputation calculation.

2. Event Effectiveness

Event effectiveness indicates that in the reputation value calculation, the weights of positive and abnormal events are different. The weight of the negative events decreased as the number of negative feedbacks increased, whereas that of the positive events increased as the number of negative feedbacks increased. In this study, the weighted sum of negative event numbers is used and, according to the Pearl growth curve function, the effectiveness of negative event θ is obtained, whereas the effectiveness of positive event 1−θ is:

(6) {θ=μ1+eErrx→jErrx→j=1∑x∈Xδx→j∑x∈Xδx→jerrx→j

where μ is the adjustment factor of the growth curve, which determines the value range of θ. $Err_{x\to j}$ is the weighted average of the abnormal event numbers; X represents the set of vehicle nodes that have interacted with a vehicle node; $\delta_{x\to j}$ represents the direct opinion weight between two vehicle nodes; and $err_{x\to j}$ represents the number of abnormal events in the interaction between the two vehicle nodes.

3. Direct Opinions Based on Event Effectiveness

Through the effectiveness of negative and positive events, the weighted vehicle node pairs’ positive event number $\alpha_{{}_{i\to j}}^{\theta }$ and the weighted vehicle node pairs’ negative event number $\beta_{{}_{i\to j}}^{\theta }$ were obtained:

(7) $$\left\{ \begin{array}{l} \alpha_{i\to j}^{\theta }=(1-\theta )\alpha_{i\to j}\\ \beta_{i\to j}^{\theta }=\theta \beta_{i\to j} \end{array}\right.$$

where $\alpha_{{}_{i\to j}}$ represents the number of positive events between vehicle nodes, and $\beta_{{}_{i\to j}}$ represents the number of negative events between vehicle nodes.

Subsequently, according to the evidence-mapping operator of SL, a direct opinion is obtained $\omega_{i\to j}=(T_{i\to j},D_{i\to j},I_{i\to j})$:

(8) $$\left\{ \begin{array}{c} T_{i\to j}=(1-I_{i\to j})\frac{\alpha_{i\to j}^{\theta }}{\alpha_{i\to j}^{\theta }+\beta_{i\to j}^{\theta }}\\ D_{i\to j}=(1-I_{i\to j})\frac{\beta_{i\to j}^{\theta }}{\alpha_{i\to j}^{\theta }+\beta_{i\to j}^{\theta }}\\ I_{i\to j}=1-\ln(1+SIM_{i\to j}) \end{array}\right.$$

In the formula, $T_{i\to j}$, $D_{i\to j}$, $I_{i\to j}$ are the parameters representing Trust, Distrust, and Indeterminacy in i’s opinion to j.

#### 4.1.3. Direct Opinion Combination Based on Three Weights and Event Validity

Subsequently, based on the TWSL algorithm [31,32] and considering the direct opinion weight, the direct combined opinion $\omega_{j}^{\mathrm{dir}}=(T_{j}^{\mathrm{dir}},D_{j}^{\mathrm{dir}},I_{j}^{\mathrm{dir}})$ is obtained:

(9) $$\left\{ \begin{array}{l} T_{j}^{\mathrm{dir}}=\frac{1}{\sum_{x\in X}\delta_{x\to j}}\sum_{x\in X}\delta_{x\to j}T_{x\to j}\\ D_{j}^{\mathrm{dir}}=\frac{1}{\sum_{x\in X}\delta_{x\to j}}\sum_{x\in X}\delta_{x\to j}D_{x\to j}\\ I_{j}^{\mathrm{dir}}=\frac{1}{\sum_{x\in X}\delta_{x\to j}}\sum_{x\in X}\delta_{x\to j}I_{x\to j} \end{array}\right.$$

where *X* represents the set of vehicles that interact with vehicle node j.

### 4.2. Indirect Combined Opinions

The blockchain stores direct interaction information between vehicles in the system. It is necessary to obtain indirect opinion paths using an algorithm and calculate the indirect combined opinions using an indirect weight formula.

#### 4.2.1. Indirect Opinion Path Search Algorithm Based on Depth-First Search

Using an indirect opinion path search algorithm based on Depth-First Search (DFS), we can obtain the required indirect opinions for the discounting operator using SL. The Algorithm 1 process is as follows:
**Algorithm 1:** Indirect Opinion Path Search Algorithm1: Initialization 2: **Input**: Opinion set G of nodes, Complete set of nodes V, Target node Vt. 3: **Output**: All indirect opinion paths Ws reaching the target node Vt. 4: **for** all element Vs //Consider nodes in set V, excluding Vt, as source nodes Vs 5:  Ws = [];     //Define the path set Ws for Vs. 6:  Opinion_Walk(G,Vs,Vt)  //Recursively search for vehicles that have interactions 7:  **if** Vs ≠ Vt and G[Vs]! = [] 8:   W←Vs;        //Add qualifying nodes to the path W 9:  **for** node in G[Vs] 10:  **if** node not in W and len(W) < 3 11:   Opinion_Walk(G,node,Vt); 12:   **if** len(W) == 3 and Vs == Vt //If an indirect path is found, add it to the path set Ws 13:   Ws←W;       //Add qualifying nodes to the path W 14:   **end if** 15:  **end if** 16:  **end for** 17:  **end if** 18: **end for** 19: **return** Ws 20: **END**

#### 4.2.2. Indirect Combined Opinions Based on Discounting Operator and Indirect Weights

By calculating indirect opinions based on Algorithm 1 and the discounting operator using SL, we can then compute the combined indirect opinions based on indirect weights.

#### 4.2.3. Indirect Opinion Based on Discounting Operator

After determining the opinion path using the indirect opinion path search algorithm, the indirect opinion definition based on the indirect path can be calculated using the discounting operator of the SL trust model. Let the opinion of node *A* on node *B* be $\omega_{A\to B}=(T_{A\to B},D_{A\to B},I_{A\to B})$ and the opinion of node *B* on node *C* be $\omega_{B\to C}=(T_{B\to C},D_{B\to C},I_{B\to C})$. The indirect opinions of both nodes *C* is defined as $\omega_{C}^{A:B}=(T_{C}^{A:B},D_{C}^{A:B},I_{C}^{A:B})$:

(10) $$\left\{ \begin{array}{l} T_{C}^{A:B}=T_{A\to B}T_{B\to C}\\ D_{C}^{A:B}=T_{A\to B}D_{B\to C}\\ I_{C}^{A:B}=D_{A\to B}+I_{A\to B}+T_{A\to B}I_{B\to C} \end{array}\right.$$

#### 4.2.4. Indirect Weights

The indirect weights $\delta_{C}^{A:m}$ are obtained according to the weight transfer formula in reference as

(11) $$\delta_{C}^{A:m}=\frac{T_{A\to m}\delta_{m\to C}}{\sum_{n\in N}T_{A\to n}\delta_{n\to C}}$$

where m represents the intermediate nodes that meet the serial relationship and N represents the vehicle node set meeting the serial relationship obtained by the indirect opinion path search algorithm.

#### 4.2.5. Indirect Combined Opinions

Based on indirect opinions and indirect weights, the weighted average was used to obtain the indirect combined opinion $\omega_{C}^{\mathrm{ind}}=(T_{C}^{\mathrm{ind}},D_{C}^{\mathrm{ind}},I_{C}^{\mathrm{ind}})$:

(12) $$\left\{ \begin{array}{l} T_{C}^{\mathrm{ind}}=\frac{\sum_{n\in N}\sum_{m\in M}\delta_{C}^{n:m}T_{C}^{n:m}}{\sum_{n\in N}\sum_{m\in M}\delta_{C}^{n:m}}\\ D_{C}^{\mathrm{ind}}=\frac{\sum_{n\in N}\sum_{m\in M}\delta_{C}^{n:m}D_{C}^{n:m}}{\sum_{n\in N}\sum_{m\in M}\delta_{C}^{n:m}}\\ I_{C}^{\mathrm{ind}}=\frac{\sum_{n\in N}\sum_{m\in M}\delta_{C}^{n:m}I_{C}^{n:m}}{\sum_{n\in N}\sum_{m\in M}\delta_{C}^{n:m}} \end{array}\right.$$

where N and M represent the vehicle node set that satisfies the serial relationship obtained by the indirect opinion path search algorithm, and $\delta_{C}^{n:m}$ represents the indirect weight obtained from the serial relationship.

### 4.3. Fusion of Opinions and System Reputation Value

By using the consensus operator of the SL trust model, direct and indirect opinions are combined to obtain the final system opinion. Supposing that the system’s direct combined opinion on node C is $\omega_{C}^{\mathrm{dir}}=(T_{C}^{\mathrm{dir}},D_{C}^{\mathrm{dir}},I_{C}^{\mathrm{dir}})$, and the indirect combined opinion on node *C* is $\omega_{C}^{\mathrm{ind}}=(T_{C}^{\mathrm{ind}},D_{C}^{\mathrm{ind}},I_{C}^{\mathrm{ind}})$, the fusion opinion on node *C* is derived from both and is denoted as $\omega_{C}^{\mathrm{dir},\mathrm{ind}}=(T_{C}^{\mathrm{dir},\mathrm{ind}},D_{C}^{\mathrm{dir},\mathrm{ind}},I_{C}^{\mathrm{dir},\mathrm{ind}})$:

(13) $$\left\{ \begin{array}{l} T_{C}^{\mathrm{dir},\mathrm{ind}}=(T_{C}^{\mathrm{dir}}I_{C}^{\mathrm{ind}}+T_{C}^{\mathrm{ind}}I_{C}^{\mathrm{dir}})/k\\ D_{C}^{\mathrm{dir},\mathrm{ind}}=(D_{C}^{\mathrm{dir}}I_{C}^{\mathrm{ind}}+D_{C}^{\mathrm{ind}}I_{C}^{\mathrm{dir}})/k\\ I_{C}^{\mathrm{dir},\mathrm{ind}}=(I_{C}^{\mathrm{dir}}I_{C}^{\mathrm{ind}})/k\\ k=I_{C}^{\mathrm{dir}}+I_{C}^{\mathrm{ind}}-I_{C}^{\mathrm{dir}}I_{C}^{\mathrm{ind}} \end{array}\right.$$

In summary, based on the SL algorithm, the final system reputation value for vehicle $E_{c}$ is

(14) $$E_{c}=T_{C}^{\mathrm{dir},\mathrm{ind}}+\gamma I_{C}^{\mathrm{dir},\mathrm{ind}}$$

where γ is a given constant given by vehicles, which indicates the uncertainty effect level on reputation for vehicles. This constant can be set as 0.5 by default [32].

## 5. Simulation and Results

### 5.1. System Setup

In this study, the performance of the proposed MSMWSL scheme is evaluated using a real-world Chongqing taxi dataset provided by Chongqing China Transport Telecommunications and Information Technology Co., Ltd., Beijing, China. This dataset comprises the movement trajectories of approximately 1000 urban taxis over a month and mainly includes data such as latitude, longitude, speed, direction, and time. This study randomly selects 100 taxis as examples. In urban areas, vehicles typically follow familiar routes within specified time periods such as similar trajectories from home to work during the day. En route, vehicles can perceive road conditions within approximately a 200-m range using equipment such as radar and sensors [39]. Consequently, vehicles can share this data to receive rewards or access this information to facilitate safe and convenient driving. 

Malicious vehicles temporarily behave normally in the initial stages to gain the trust of other vehicles. This study assumes that after the detection period begins, they exhibit abnormal behavior. To identify these vehicles, the system periodically calculates their reputation values, and prohibits vehicles with reputation values below a certain threshold from accessing the network. We compared the proposed MSMWSL algorithm with the TWSL [31,32] and SL algorithms. The initial reputation value of all vehicles is represented by $E_{0}=0.6$ [35], and the reputation threshold sets to 0.5 [31,32].

### 5.2. Simulation Experiment Parameters

The parameter settings for the simulation experiment are shown in Table 1 [31].

### 5.3. Simulation Results and Analysis

**Figure 3:**
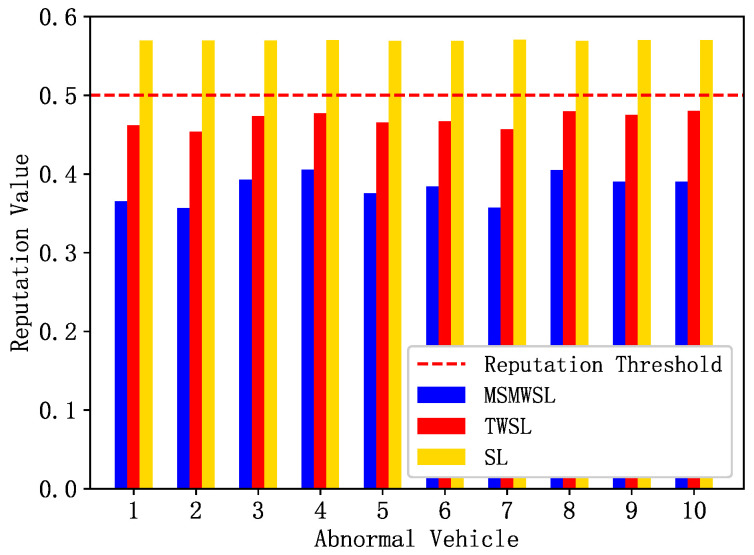
Comparison of reputation values of 10 abnormal vehicles under 3 algorithms.

As shown in Figure 3, ten malicious vehicles were randomly selected for reputation updates during the observation period, and all vehicles interacted randomly with other vehicles. Figure 3 shows that the reputation values of all malicious vehicles were below the initial reputation value of 0.6. Additionally, the reputation values of the malicious vehicles under the MSMWSL and TWSL algorithms were all below the reputation threshold. Notably, the reputation values of the malicious vehicles under the MSMWSL algorithm were significantly lower than those under the TWSL and SL algorithms. This is because the MSMWSL algorithm considers various factors such as event validity and the three weights of direct opinions and integrates both direct and indirect opinion sources. This allows the system to calculate reputation more accurately, thereby identifying malicious vehicles more quickly.

**Figure 4:**
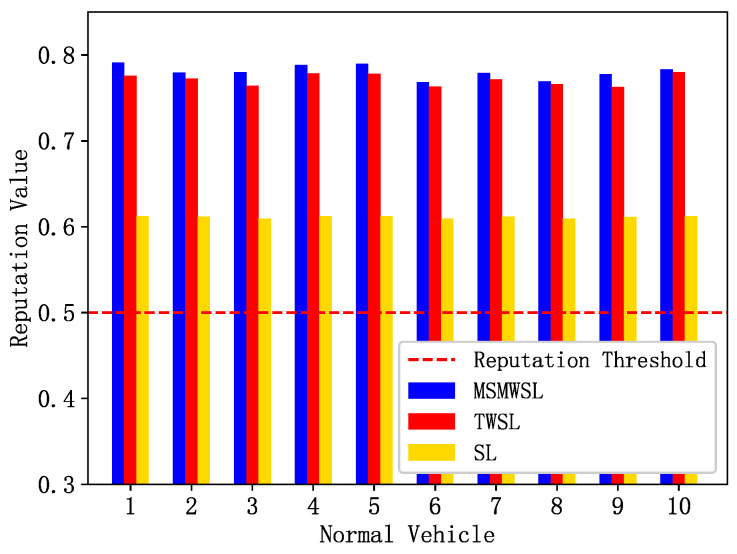
Comparison of reputation values of 10 normal vehicles under 3 algorithms.

As shown in Figure 4, during the observation period, ten normal vehicles were randomly selected for reputation updates, with vehicles interacting randomly with each other. In Figure 4, the reputation values of all the normal vehicles are above the initial reputation value of 0.6. Moreover, the calculated results of the MSMWSL algorithm were generally higher than those of the TWSL and SL algorithms. This is because all reputation calculation values are sufficiently weighted by various factors, allowing vehicles to choose vehicles with higher reputations intuitively when sharing information. This also indicates that, while quickly lowering the reputation values of malicious vehicles, the MSMWSL algorithm still performs well in evaluating the reputation of normal vehicles.

**Figure 5:**
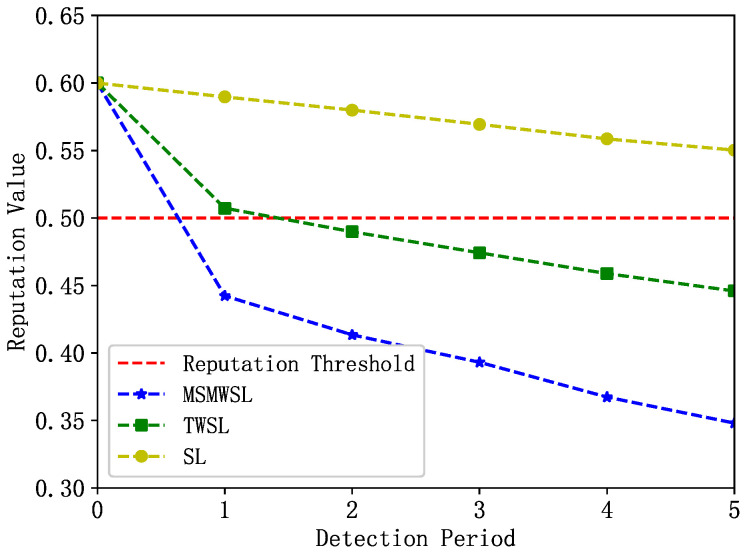
Change of abnormal vehicle reputation value with detection period under 3 algorithms.

The change in the reputation value of an abnormal vehicle with the number of detection cycles is shown in Figure 5. Initially, this abnormal vehicle tended to provide high-quality data to other vehicles to gain trust in the system. However, during the detection window, the vehicle’s reputation value gradually decreases owing to abnormal behavioral events. As shown in Figure 5, the reputation value of the abnormal vehicles under the MSMWSL and TWSL algorithms declined significantly faster than that under the SL algorithm. After the first detection cycle, the reputation values calculated by the MSMWSL algorithm decreased below the reputation threshold of 0.5, and the MSMWSL algorithm’s value was lower than that of the TWSL algorithm. However, the values for the TWSL and SL algorithms remained above the reputation threshold. Eventually, in all detection cycles, the reputation value of the abnormal vehicles calculated using the MSMWSL algorithm was significantly lower than that calculated using the other algorithms. Overall, the MSMWSL algorithm outperformed the other algorithms and exhibited higher detection efficiency for abnormal vehicles.

**Figure 6:**
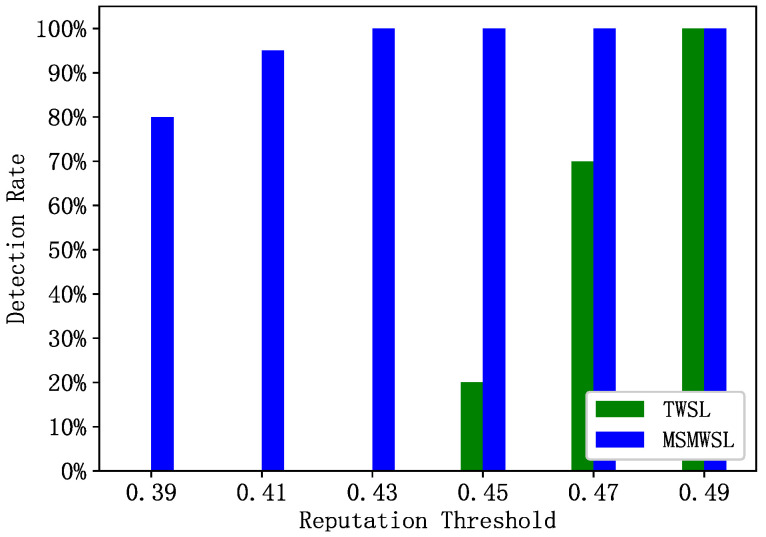
Abnormal vehicle detection rates of 2 algorithms under different reputation thresholds.

During the observation period, 30 abnormal vehicles were randomly selected for reputation updates, and their detection rates were compared using the two algorithms and six reputation threshold values, as shown in Figure 6. This figure illustrates a comparison of the abnormal vehicle detection rates within the same detection cycle for the MSMWSL and TWSL algorithms. It is evident from Figure 6 that under different threshold values, the MSMWSL algorithm has higher detection rates for abnormal vehicles than the TWSL algorithm. With a reputation threshold of 0.39, the detection rate of the MSMWSL algorithm for abnormal vehicles reached 80% and increased to 100% when the threshold was 0.43. By contrast, the TWSL algorithm has detection rates of 20% and 70% for abnormal vehicles at reputation thresholds of 0.45 and 0.47, respectively, and reaches 100% when the threshold is 0.49.

This is because the MSMWSL algorithm can reduce the reputation values of misbehaving vehicles more rapidly under different thresholds than other algorithms, allowing for faster differentiation between abnormal and normal vehicles. Consequently, this algorithm can effectively eliminate potential security threats and enhance the safety of information sharing within a network.

**Figure 7:**
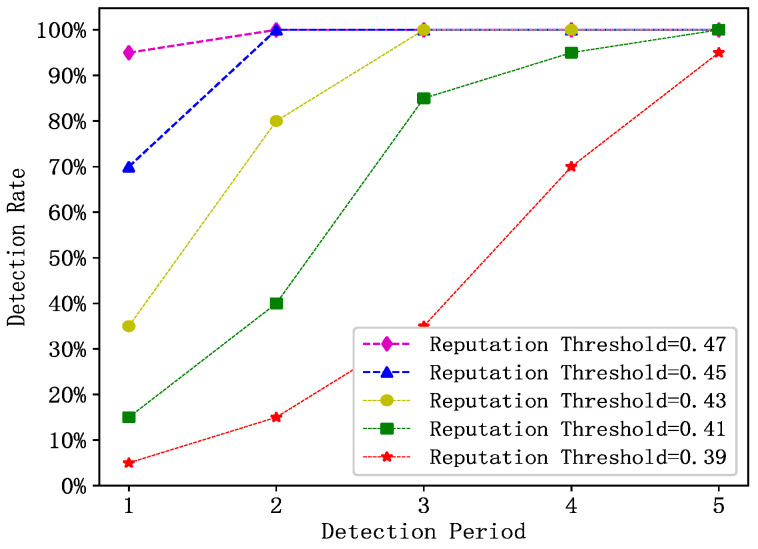
Variation in abnormal vehicle detection rate with period and reputation thresholds using MSMWSL algorithm.

The changes in the detection rates of 30 randomly selected abnormal vehicles for reputation updates during the observation period under different reputation thresholds in the MSMWSL algorithm are shown in Figure 7. It should be noted that the higher the reputation threshold, the stronger the ability to differentiate abnormal vehicles.

At a reputation threshold of 0.47, the MSMWSL algorithm achieved a 95% detection rate for abnormal vehicles in the first detection cycle, and reached 100% in the second cycle. When the reputation threshold was 0.45, the MSMWSL algorithm had an identification rate of 70% for abnormal vehicles in the first detection cycle, which increased to 100% in the second cycle. With a reputation threshold of 0.43, the MSMWSL algorithm attained an 80% identification rate for abnormal vehicles in the second detection cycle and achieved 100% detection in the third cycle. At a reputation threshold of 0.41, the MSMWSL algorithm achieved an 85% identification rate of abnormal vehicles in the third detection cycle. When the reputation threshold was 0.39, the MSMWSL algorithm achieved a 70% identification rate for abnormal vehicles in the fourth detection cycle.

In summary, when the reputation threshold is lower than the typical value of 0.5, the MSMWSL algorithm can achieve a high detection rate for abnormal vehicles. This is attributed to the MSMWSL algorithm proposed in this study, which considers various factors and integrates multisource information. This allows for the rapid identification of abnormal vehicles in each detection cycle under different reputation values, thereby enhancing the overall defensive capabilities of the system against abnormal vehicles.

## 6. Conclusions

To address trust and security risks in the data-sharing process of IoV, this paper proposes a secure, blockchain-assisted reputation management scheme. First, it employs a VEC model to address issues such as limited vehicle resources and task latency. It then leverages the consortium blockchain technology to establish a blockchain network among edge servers, ensuring the security and authenticity of data storage at a reasonable cost. Next, to defend against malicious attacks from abnormal vehicles and prevent a group of such vehicles from unfairly influencing the reputation of the target vehicle through biased feedback, the method utilizes asymmetric encryption techniques provided by the blockchain to prevent vehicles from being continuously identified and attacked. Moreover, when calculating reputation values, this study employs an SL trust model to compute reputation values based on feedback events during the car-sharing process, taking into account factors such as familiarity, timeliness, and trajectory similarity to sufficiently weight opinions. The discounting and consensus algorithms of the SL trust model are then used to obtain a reputation value that combines direct and indirect opinions, improving the multisource nature and accuracy of the reputation value calculation. Finally, to counteract false message attacks and prevent abnormal vehicles from broadcasting false messages that disrupt other vehicles, this study calculates the effectiveness of both positive and negative events using historical interaction records and a Pearl model based on growth curve functions, in addition to considering multi-source opinions. By weighting the interaction events with the effectiveness of the two types of events, the vehicle reputation calculation becomes more precise.

In conclusion, this study achieved accurate reputation calculations and ensured high-quality data sharing. Using the proposed algorithm, the system can identify and eliminate the security risks posed by abnormal vehicles, and vehicles can efficiently select the best data providers. The experimental results demonstrate that this method has significant advantages in terms of improving the detection rate of abnormal vehicles and ensuring the security of data sharing.

## Figures and Tables

**Table 1:** Simulation experiment parameters table.

Parameter Name	Value
Vehicle Count	100
Message Frequency	[5,10] times/cycle
Timeliness Parameter	η=10,ε=1.05
Trajectory Similarity Weight	$\tau_{1}=0.5,\tau_{2}=0.5$
Direct Opinion Weight	$\rho_{1}=0.3,\rho_{2}=0.3,\rho_{3}=0.4$
Pearl Growth Curve Function Adjustment Factor	μ=0.8

## Data Availability

Not applicable.
